# Antioxidant, Photoprotective, and Antimicrobial Potential of Oil Extract of *Usnea barbata* L. F.H.Wigg from Călimani Mountains, Romania

**DOI:** 10.3390/molecules31081324

**Published:** 2026-04-17

**Authors:** Mihaela Afrodita Dan, Marina Ionela Nedea, Emma Adriana Ozon, Anca Ungurianu, Andreea Roxana Ungureanu, Violeta Popovici, Adina Magdalena Musuc, Veronica Bratan, Radu George Cazacincu, Andreea Letiția Arsene, Dumitru Lupuliasa, Denisa Margina

**Affiliations:** 1 Faculty of Pharmacy, “Carol Davila” University of Medicine and Pharmacy, 020945 Bucharest, Romania; mihaela.dan@umfcd.ro (M.A.D.); marina.nedea@umfcd.ro (M.I.N.); emma.budura@umfcd.ro (E.A.O.); anca.ungurianu@umfcd.ro (A.U.); andreea.arsene@umfcd.ro (A.L.A.); dumitru.lupuliasa@umfcd.ro (D.L.); denisa.margina@umfcd.ro (D.M.); 2 Department of Pharmacy, Sanador Clinical Hospital, 010991 Bucharest, Romania; andreea.ungureanu@sanador.ro; 3 Center for Mountain Economics, “Costin C. Kiritescu” National Institute of Economic Research (INCE-CEMONT), Romanian Academy, 725700 Vatra-Dornei, Romania; 4 Institute of Physical Chemistry—Ilie Murgulescu, Romanian Academy, 060021 Bucharest, Romania; vbratan@icf.ro; 5 Faculty of Pharmacy, Ovidius University of Constanta, 900470 Constanta, Romania; radu.cazacincu@univ-ovidius.ro

**Keywords:** *Usnea barbata*, Jojoba oil, Peppermint essential oil, Vitamin E, antioxidant activity, photoprotective effect, antimicrobial potential

## Abstract

In order to enhance the value and stability of vegetable oils, they may be enriched with essential oils and plant extracts with strong antioxidant activity, yielding innovative formulations with potential applications in skincare. The present research aims to investigate the bioactive properties of an *Usnea barbata* extract in Jojoba oil (JO) enriched with 5% Peppermint oil (PEO), and 10% vitamin E (PJO). The oil extract (UBPJO) was obtained through cold maceration. The UBPJO antioxidant activity was investigated using ABTS (2,2′-azino-bis(3-ethylbenzothiazoline-6-sulfonic acid)) and DPPH (2,2-diphenyl-1-picrylhydrazyl)-based methods. The sunscreen capacity of UBPJO was evaluated by measuring their sun protection factor (SPF) values. The antimicrobial potential was investigated against *S. aureus*, *E. coli*, and *C. albicans.* The antioxidant activity of UBPJO was 1.5 times higher than that of PJO. Consequently, the sunscreen efficacy (assessed by SPF measurements) indicated satisfactory UVB protection abilities against *S. aureus* (UBPJO vs. PJO: 32.41 vs. 30.58). UBPJO exhibited significantly greater inhibitory effects compared to PJO against *S. aureus* (MIC = 18.75 ± 6.25 vs. 37.50 ± 0.00, *p* < 0.05). and *C. albicans* (9.62 ± 2.87 vs. 37.5 ± 12.5, *p* < 0.05). The bioactive properties investigated in the present study support the inclusion of UBPJO in various skin protective formulations with antiaging, antibacterial, and antifungal effects.

## 1. Introduction

Achieving and maintaining healthy skin involves a comprehensive approach that incorporates daily protection, targeted skincare routines, and mindful lifestyle choices [1]. One of the most significant contributors to visible skin aging is UV exposure, which accounts for 80–90% of the signs of aging [2,3,4].

Exposure to ultraviolet (UV) radiation is a major stress factor for the skin, directly affecting epidermal cellular structure and function and often causing various skin cancers. Ultraviolet B (UVB) radiation, with wavelengths between 280 and 320 nm, triggers an acute inflammatory response of the skin, mainly mediated by proinflammatory substances derived from arachidonic acid (prostaglandins and leukotrienes) [5]. It is mainly responsible for erythema, skin alterations, and even carcinogenesis [6,7,8,9]. On the other hand, UVA radiation, spanning 320–400 nm, penetrates more deeply, leading to the development of skin photoaging, characterized by reduced elasticity, skin tone loss, collagen breakdown, and wrinkles [10,11].

According to literature data, chronic exposure to UV radiation results in oxidative stress via ROS generation, DNA damage, hyperpigmentation, premature photoaging, and increases the long-term risk of melanoma and non-melanoma skin cancers [12,13]. Therefore, making daily sun protection a non-negotiable part of a skincare regimen is essential. This can include using a broad-spectrum sunscreen with at least Sun Protection Factor (SPF) of 30, as well as incorporating antioxidants, moisturizers, and antimicrobials, which can enhance overall skin health and resilience against aging [14,15]. Most commercial formulations contain chemical or mineral filters that can cause irritation, sensitization, or environmental harm [16,17]. Therefore, modern consumers are increasingly aware of the potential health risks posed by synthetic chemicals in personal care products [18,19], driving a significant shift toward natural, eco-friendly cosmetic formulations. This trend has spurred extensive scientific research into plant-derived compounds that can serve as functional ingredients in cosmetics—offering not only aesthetic benefits but also health-promoting effects, including sunburn protection and antioxidant and antimicrobial properties [20].

Various cosmetic ingredients can be identified using the International Nomenclature of Cosmetic Ingredients (INCI), which provides a standardized system to ensure clarity and consistency in the cosmetics industry. It contains over 30,000 INCI names, chemical structures, synonyms, and regulatory references. The International Cosmetic Ingredient Dictionary and Handbook, where these names are listed, is also available electronically as wINCI, making it easily accessible for those in the field. This system enhances transparency and fosters trust among consumers and manufacturers alike, and is available online at https://www.personalcarecouncil.org/resources/inci/, accessed on 13 April 2026. The European Commission official Database (CosIng, available online at https://ec.europa.eu/growth/tools-databases/cosing/, accessed on 13 April 2026) includes over 15,000 ingredients and provides details on the INCI name, CAS number, ingredient functions, and possible restrictions on use under Regulation (EC) No 1223/2009. As an initiative of the European cosmetics industry, COSMILE Europe provides transparent, easy-to-understand information for consumers about the safety and role of each ingredient, available online at https://cosmileeurope.eu/. It contains approximately 30,000 cosmetic ingredients. A plant-derived product with multiple benefits for skincare, included in the above-mentioned databases, is a lichen extract (*Usnea barbata* extract) available online at https://cosmileeurope.eu/inci/detail/16750/usnea-barbata-extract/, accessed on 13 April 2026. Over six years (2016–2022), as part of a doctoral and postdoctoral project, our team investigated the composition and bioactivities of *U. barbata* harvested from an unpolluted zone in the Calimani Mountains. *U. barbata* extracts were obtained in various solvents (ethanol, methanol, ethyl-acetate, acetone, and Canola oil) through different methods (maceration and Soxhlet extraction). Usnic acid and other phenolic secondary metabolites were identified and quantified through Ultra-High-Performance Liquid Chromatography (UHPLC): caffeic acid, p-coumaric acid, ellagic acid, chlorogenic acid, cinnamic acid, and gallic acid [21,22]. We also isolated usnic acid from the dry *U. barbata* extract in ethyl acetate by semi-preparative chromatography [23], and validated a UHPLC method for the determination of usnic acid content in *U. barbata* extract in Canola oil [24]. Moreover, the most active extracts were incorporated in mucoadhesive oral films with potential applications in oral cancer [25].

Based on our previously published results, *U. barbata* harvested from the same unpolluted zone of the Calimani Mountains in the same period was extracted in a mixed oil solvent, Jojoba oil (JO) enriched with 5% Peppermint essential oil (PEO) and 10% vitamin E. We recently identified the bioactive constituents (using the GC-MS method), determined the total phenolic content (including usnic acid and other phenolic acids), and investigated the physicochemical properties of this complex oil extract, with potential applications in the cosmetic field [26]. Our published results indicated that the combination of all four ingredients (*Usnea barbata* extract in Jojoba oil, enriched with 10% Vitamin E and 5% Peppermint oil, UBPJO) creates a multifunctional cosmetic system in which each ingredient contributes complementary properties (Table 1) [26].

The addition of *U. barbata* extract to the already antioxidant-rich base of Jojoba oil, Peppermint oil, and Vitamin E significantly increased the total phenolic content. This suggests a true additive-to-synergistic enhancement of antioxidant capacity, because:

○ Vitamin E (tocopherol) contributes lipid-soluble radical-scavenging activity
○ Peppermint oil contributes terpenes with antioxidant properties
○ Jojoba oil provides a stable, oxidation-resistant liquid wax matrix
○ *U. barbata* is rich in unique lichen secondary metabolites with phenolic structure (e.g., usnic acid, depsides, depsidones) that substantially boost the total phenolic content [39].

Atomic Force Microscopy (AFM) and Fourier Transform Infrared (FTIR) spectroscopy confirmed the physicochemical compatibility of the combined ingredients [26]:

○ AFM revealed surface roughness differences between PJO and UBPJO, indicating that *U. barbata* extract integrates into the oil matrix and modifies its microstructure;
○ FTIR confirmed the chemical integrity of the combined formulation, showing no destructive interactions between components.

Therefore, the combination has a higher oxidative stability [26], suggesting that *U. barbata* phenolics synergize with Vitamin E to quench free radicals more effectively. Jojoba oil’s inherent stability (as a liquid wax ester rather than a triglyceride) provides a protective matrix that slows oxidative chain reactions [40,41], and Peppermint oil’s volatile antioxidants contribute an additional layer of protection [29,42].

This complex formulation also showed improvements in rheological properties [26]. These aspects are relevant because they affect the bioavailability of active compounds at the skin surface—a more spreadable formulation ensures better contact time and penetration of the antioxidant, antimicrobial, and UV-protective compounds.

All previous findings support the conclusion that the four ingredients work synergistically rather than merely additively, making the combined formulation a promising candidate for natural, multifunctional skincare products.

As an innovative approach in developing natural skin care products, UBPJO-based formulations are expected to combine emollient, antioxidant, and antimicrobial effects with slightly enhanced UV protection. Thus, the present study aims to investigate the antioxidant activity, sunscreen properties, and antimicrobial potential of *U. barbata* extract in JO enriched with 5% Peppermint oil and 10% vitamin E.

## 2. Results

### 2.1. Antioxidant Activity

To accurately evaluate the antioxidant activity of PJO and UBPJO, the samples were diluted with methanol (1:1, 1:5, 1:10, and 1:25) and compared with a solvent-based standard to evaluate dose–response behavior.

#### 2.1.1. DPPH Method

**Figure 1:**
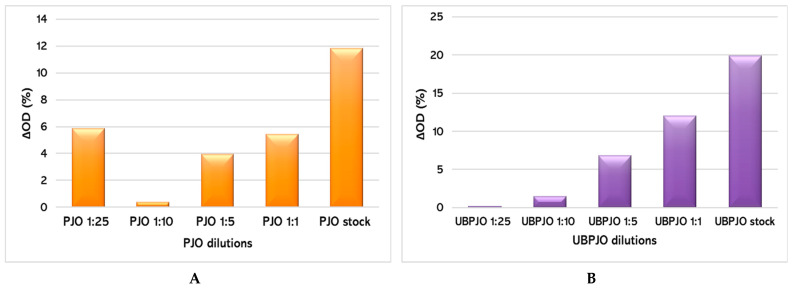
Antioxidant capacity of various dilutions of PJO (**A**) and UBPJO (**B**); ΔOD—decrease in optical density; PJO—Jojoba oil with 5% Peppermint Essential Oil and 10% Vitamin E; UBPJO—*U. barbata* oil extract.

The in vitro radical-scavenging capacity of PJO and UBPJO was assessed using the DPPH method (Figure 1).

The highest PJO dilution (1:25) has the second-most intense antioxidant effect after the stock solution (ΔOD = 5.87% vs. 11.87%, Figure 1A), suggesting a U-shape concentration-dependent radical scavenging capacity.

Our results show that the standard OD dynamics was 4,74%. For the UBPJO, ΔOD (%) was almost insignificant at the highest dilutions (0,19% for the 1:25 dilution and 1,50% for the 1:10 dilution), but became highly significant for the 1:5, 1:1, and undiluted samples. (6,87%, 12,03%, and 19,92% respectively). This indicates a concentration-dependent radical scavenging capacity. Overall, the ΔOD values for UBPJO were 1.5 times higher than PJO (Figure 1A,B).

#### 2.1.2. ABTS Method

For the ABTS antioxidant capacity assay, the same dilutions (1:1, 1:5, 1:10, and 1:25 in methanol) were employed. We were unable to assess the undiluted samples because an emulsification process rendered them unsuitable for reading at the specified OD. The literature indicates that the complex solubility of oil extracts with multiple components can skew results in ABTS assays (BenchChem Technical Support).

**Figure 2:**
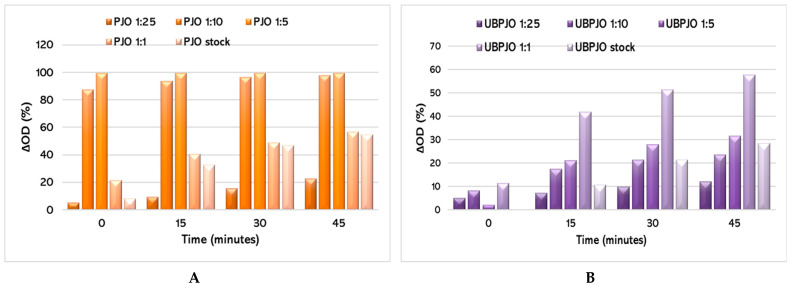
ABTS radical scavenging capacity of PJO (**A**) and UBPJO (**B**) dilutions; ΔOD—decrease in optical density; PJO—Jojoba oil with 5% Peppermint Essential Oil and 10% Vitamin E; UBPJO—*U. barbata* oil extract.

Results from the ABTS method show that the 1:1 dilution of UBPJO has the strongest antioxidant effect among all dilutions, while PJO is also highly active at 1:5 and 1:10, consistently with no significant differences (Figure 2A). Furthermore, for all dilutions, the antioxidant capacity of PJO was approximately twice as high as that of UBPJO dilutions (Figure 2A,B).

For UBPJO, the most pronounced effect occurred at the 1:1 dilution, while higher dilutions (1:5 to 1:25) showed progressively flatter responses, suggesting a dilution-dependent decrease in antioxidant capacity (Figure 2B).

This pattern might indicate potent antioxidant activity, best captured at moderate dilution, where solubility issues in the undiluted oil are resolved without excessive dilution that masks the effect.

The ABTS results show an optimal 1:1 dilution for the UBPJO, where maximum radical scavenging coincides with greater solubility in alcohol (Figure 2B). This highlights the limitations of the conventional ABTS assays for oil-based samples. In undiluted samples, the interaction between lipophilic antioxidants (e.g., tocopherols) and the hydrophilic ABTS^+^ radical is restricted, leading to underestimated activity, particularly because emulsification occurred.

### 2.2. Sunscreen Properties

Table 2 and Table 3 present the SPF results for PJO and UBPJO at different dilutions obtained using the spectrophotometric method. The calculated SPF for UBPJO was slightly higher than that for PJO (32.41 vs. 30.58).

**Figure 3:**
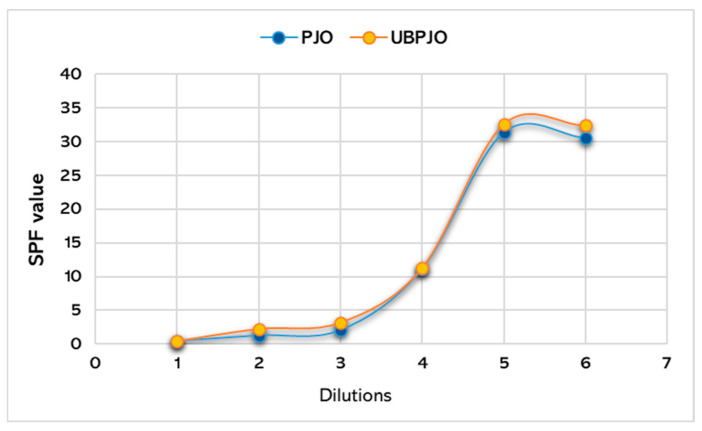
Variation in SPF values, in the wavelength range of 290–320 nm at 5 nm intervals, for PJO and UBPJO; PJO—Jojoba oil with 5% Peppermint Essential Oil; UBPJO—*U. barbata* oil extract. Dilutions 1–6 = 1:10; 1:5; 1:4; 1:2; 1:1; stock solution.

The results obtained for SPF according to the equation developed by Mansur [43] are shown comparatively in Figure 3.

### 2.3. Antimicrobial Activity

The results of the antimicrobial activity assessment focused on two areas: determining the minimum inhibitory concentration (MIC) and evaluating the ability of PJO and UBPJO to inhibit microbial biofilm formation. To assess oil samples, we used a 20% Tween 80 solution in ethanol for solubilization, allowing the compounds to disperse in the aqueous culture medium. Tween 80 was included as a positive control in the microbiological analyses. All determined MIC values (mg/mL) were illustrated in Table 4, compared with those of conventional antibiotics (µg/mL) reported by Rankovic et al. for the same ATCC pathogen strains, which were used to investigate the antimicrobial potential of the *U. barbata* acetone extract [44].

Data from Table 4 show that UBPJO is significantly more effective than PJO against *S. aureus* and *C. albicans*, as evidenced by lower MIC values (*p* < 0.05). On *E. coli*, T80 has the highest inhibitory effect (*p* > 0.05).

To further characterize their biological effects, the influence of PJO and UBPJO on microbial adherence to an inert substrate was subsequently investigated at subinhibitory concentrations (MIC/2 and MIC/4), as an indicator of their potential effect on early biofilm formation.

**Figure 4:**
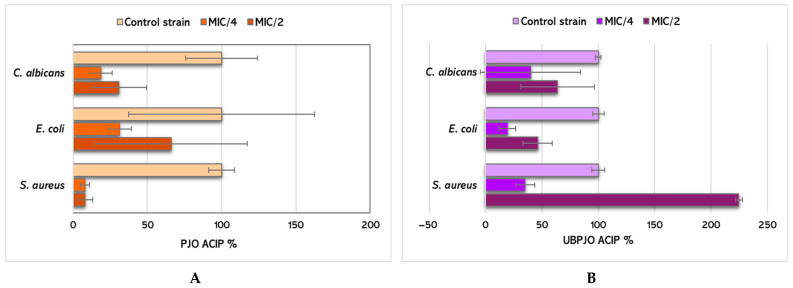
Antibiofilm activity: Adherence capacity inhibition percentage (ACIP %) for PJO (**A**) and UBPJO (**B**) against tested microorganisms.

Figure 4A,B illustrate the adherence capacity inhibition percentage (ACIP) of PJO and UBPJO against the tested microorganisms at subinhibitory concentrations (MIC/2 and MIC/4).

PJO has an increased ability to inhibit the adhesion of *E coli* to the inert substrate than UBPJO at MIC/2 and MIC/4 (66.2 ± 51.18 and 31.34 ± 7.79 vs. 46.17 ± 12.84 and 19.25 ± 7.36, *p* > 0.05, Figure 4).

Conversely, UBPJO at MIC/2 exhibited substantial antibiofilm activity against *S. aureus* (224.63 ± 2.85 vs. 7.95 ± 5.11, *p* < 0.001). Significantly higher activity was exhibited at MIC/4 (35.01 ± 8.47 vs. 7.87 ± 3.12, *p* < 0.01, Figure 4).

Unexpectedly, UBPJO at MIC/2 yielded an ACIP value of 224.63 ± 2.85 against *S. aureus*, indicating a paradoxical response at subinhibitory concentration and suggesting that microbial adherence was not inhibited under these conditions. This finding may reflect increased crystal violet-retained surface-associated biomass rather than effective inhibition of early adherence. At MIC/4, the ACIP value was markedly lower (35.01 ± 8.47), indicating a concentration-dependent effect.

Against *C. albicans*, UBPJO at MIC/2 exhibited a higher ACIP value than PJO (63.75 ± 32.66 vs. 30.99 ± 18.42; *p* > 0.05), suggesting a moderate influence on fungal adherence.

## 3. Discussions

The current study examined a complex oil extract of *U. barbata* in Jojoba oil, enriched with 5% PEO and 10% Vitamin E, for potential skincare uses. The results show that combined actions among all ingredients yield beneficial pharmacological properties (antioxidant, photoprotective, and antimicrobial) for this innovative formulation.

Vitamin E serves as the cornerstone of antioxidant synergy in this formulation system, and its contributions are well documented [45]. It donates a hydrogen atom to stabilize free radicals generated by UV exposure, pollution, or oxidative stress [46]. However, in doing so, it becomes a tocopheroxyl radical itself—relatively stable due to its aromatic nature, but no longer active [47,48]. Vitamin E is found in lipid membranes [49] and is the first barrier to neutralize free radicals produced by UV radiation [50,51]. During this process, it becomes oxidized, forming a less active radical [52]. Vitamin E primarily functions in the lipid phase, which directly relates to its interaction with jojoba oil, as a lipid-rich carrier. Topical application of alpha-tocopherol (the active form of vitamin E) has been shown to increase collagen density during the early phase of wound healing, with a significant rise in fibroblast numbers observed in treated animals compared to placebo [53,54]. Vitamin E can also serve as a cofactor to stabilize Vitamin A and protect it from oxidation, suggesting a broader role as a molecular protector of other sensitive actives within a formulation [51,55].

Jojoba oil is not a typical triglyceride oil—its structure closely resembles that of liquid wax, which gives it excellent oxidative stability, high skin compatibility, and greater stability than regular oils [56]. This wax-like structure makes jojoba oil an ideal lipid-phase carrier for fat-soluble ingredients like Vitamin E and components of peppermint oil. The liquid wax structure of jojoba oil stabilizes lipid-soluble actives, such as Vitamin E and terpenoids from peppermint essential oil [57]. The skin compatibility of JO enhances the penetration and bioavailability of co-ingredients [58]. Since Vitamin E functions within the lipid phase, jojoba oil’s wax-like lipid matrix provides an ideal environment for Vitamin E to act as an antioxidant at the membrane level. This shows that jojoba oil not only carries Vitamin E but also creates a supportive environment for its activity. It also enhances the synthesis of collagen and hyaluronic acid and diminishes inflammation in human skin [59]. The oxidative resilience of JO decreases the oxidative degradation of sensitive ingredients [60]. Moreover, due to its emollient properties, JO creates a protective film that prolongs the contact time of antimicrobial agents [61,62].

Peppermint oil’s antimicrobial properties complement *Usnea barbata’s* secondary metabolites activity. While usnic acid and other phenolic metabolites target bacterial cell membranes and metabolic processes, peppermint oil’s terpenoids disrupt microbial membrane integrity through a different but complementary pathway, potentially broadening the spectrum of antimicrobial coverage. Menthol is a well-recognized skin penetration enhancer [63]. By temporarily modifying the lipid structure of the stratum corneum [64], peppermint oil can increase the bioavailability of vitamin E in deeper skin layers, enhance the delivery of usnic acid from *U. barbata*, and improve the overall efficacy of the formulation system [65]. Peppermint oil’s cooling and anti-inflammatory properties [66] complement Vitamin E’s documented anti-inflammatory effects [67]. Studies have shown that Vitamin E in combination with other actives leads to significant decreases in Tumor Necrosis Factor-Alpha (TNF-α) expression [68], and peppermint oil’s menthol component activates Transient Receptor Potential Cation Channel Subfamily M Member 8 (TRPM8) receptors [69], providing an additional, mechanistically distinct anti-inflammatory pathway.

*U. barbata* contains phenolic secondary metabolites with specific structures; usnic acid is its primary bioactive compound [70]. Usnic acid possesses its own antioxidant properties through phenolic hydroxyl groups that can scavenge free radicals [71]. This creates a multi-molecular antioxidant network in combination with Vitamin E, resulting in broader radical-scavenging coverage. Usnic acid disrupts bacterial DNA and RNA synthesis and membrane function [72]. When combined with peppermint oil’s membrane-disrupting terpenoids [73]. The two agents attack microbial cells via dual mechanisms, potentially reducing the minimum inhibitory concentration (MIC) required for each.

The comparative evaluation of PJO and UBPJO using DPPH and ABTS assays highlights method-dependent differences in antioxidant profiling. DPPH results indicate a clear concentration-dependent response, with UBPJO exhibiting stronger radical-scavenging activity overall, reaching values approximately 1.5 times those of PJO and showing maximal effects in less diluted or undiluted samples. In contrast, ABTS results emphasize the importance of solubility, identifying a 1:1 dilution as optimal, particularly for UBPJO, where improved dispersion enhances measurable activity. PJO displayed consistently higher activity than UBPJO in ABTS, especially at intermediate dilutions. These discrepancies reflect the physicochemical constraints of each method, particularly for lipophilic systems. Overall, DPPH proves more suitable for direct assessment of oil-based antioxidants, while ABTS offers complementary insights when appropriate dilution improves interaction conditions. The use of appropriate alcohol-based solvents can improve the dispersion of lipophilic compounds and facilitate their interaction with hydrophilic reactive species, thereby enhancing measurement reliability. From a formulation perspective, this approach is particularly valuable in skincare product development, where controlled alcoholic dilution can enhance the solubility, stability, and bioavailability of antioxidant compounds in emulsified or hydroalcoholic systems.

The determination of the sun protection factor (SPF) for vegetable oils used in skincare products is a topic of intense investigation in the literature, highlighting significant variability in results across methodologies, concentrations, and formulation types [74,75]. Sun protection factor (SPF) is a quantitative indicator used in sunscreens to assess a formulation’s photoprotective efficacy against ultraviolet (UV) radiation. In this study, both PJO and UBPJO were subjected to SPF determination by spectrophotometry. The results obtained indicated SPF values of ~30.58 for PJO and ~32.41 for UBPJO. These values demonstrate the ability of both formulations to act as natural photoprotective agents, and the smaller difference between the two confirms an increase in efficiency when using the combination with lichen extract.

In both oil samples, PJO and UBPJO, a progressive increase in absorbance towards lower wavenumbers (closer to 290 nm) was observed. This aligns with the literature, which indicates that the increased absorbance near 290 nm results from the higher-energy excitation of the conjugated π-electron system, a common absorption characteristic of phenols and other aromatic compounds, and matches the compositions of both samples determined in previous research. However, UBPJO shows slightly higher absorbance at all measured wavelengths than PJO, and this is due mainly to the synergistic interaction between the complex oil base’s bioactive compounds and the phenolic lichen secondary metabolites [76,77], including usnic acid [78]. *U. barbata* constituents ensure a higher concentration of chromophoric groups capable of absorbing UVB radiation. In addition to absorbing UV light directly, these phenolic compounds also have antioxidant properties. The dual function of these metabolites helps enhance photoprotection through two mechanisms. The absorption of UV photons (known as primary photoprotection) results in energy dissipation via non-radioactive mechanisms [79]. The active ROSs generated by UV exposure are neutralized. This secondary photoprotection is also enhanced by the presence of alpha-tocopherol (the form of vitamin E) [80,81].

Pure vegetable oils generally exhibit low intrinsic SPF values, but they have recently been widely used in sunscreen formulations due to their unique UV-absorption properties and improvements in the spreadability and skin-adsorption characteristics of the final products [82,83]. The literature reports SPF values of 6.02 for JO, 9.28 for olive oil, 18.81 for carrot seed oil, and 22.04 for wheat germ oil [84]. The SPF values of natural Jojoba oil reported in the literature are generally similar to those of other vegetable oils [85].

Montenegro et al. studied the use of 1% pomegranate and shea oil as natural UV filters to improve the SPF of sunscreen preparations [83]. Another recent study reported that the SPF of a cream base + Oksibenzon 2% + Octil metoximate 5% was significantly increased from 21.12 to 37.01 by adding 10% JO.

The SPF value of 30.58 for the PJO is consistent with literature data for vegetable oils combined with other compounds (PEO and vitamin E, as used in our research). The measured absorbance in the 290–330 nm spectral range can be attributed to the optical properties of the JO and PEO. Jojoba oil contributes to moderate UV absorption and, consequently, to a slight photoprotective activity, while PEO and vitamin E contribute to minor UV absorption and antioxidant activity.

Furthermore, the SPF value of 32.41 for UBPJO indicates that the *U. barbata* extract can moderately enhance the photoprotective performance and UV-absorbing capacity of formulations when they are incorporated into lipid-based matrices. Several studies have reported that usnic acid and other phenolic secondary metabolites of *U. barbata* exhibit antioxidant, anti-inflammatory, antiviral, and antimicrobial effects. These biological activities are particularly relevant for photoprotection because UV radiation induces oxidative stress and an anti-inflammatory skin response [86,87].

The antimicrobial activity was evaluated against the most common pathogens (*S. aureus*, *E. coli*, and *C. albicans*); our results revealed a significant antimicrobial and antibiofilm activity of UBPJO on *S. aureus* and *C. albicans.* They are similar to those of previous studies, which reported negligible inhibitory activity of *U. barbata* extracts against Gram-negative bacteria, including *E. coli* [88,89]. The antimicrobial potential of UBPJO is mainly due to the synergistic action of its 2 ingredients, *U. barbata* and PEO.

In the adherence assay, an unexpected ACIP value above 100% was observed for UBPJO against *S. aureus* at MIC/2, suggesting that subinhibitory concentrations may paradoxically alter microbial surface-associated behavior. Such responses may reflect increased biomass retention in the crystal violet assay rather than true inhibition of early biofilm formation. These findings highlight the need for caution when interpreting adherence-based endpoints at subinhibitory concentrations [90].

*U. barbata* efficacy against *S. aureus* is mainly due to phenolic secondary metabolites, especially usnic acid [22,91]. Usnic acid’s anti-staphylococcal activity involves a multitarget approach. It primarily disrupts the bacterial cell membrane, rapidly inhibits RNA, DNA, and protein synthesis, and impairs peptidoglycan/fatty acid biosynthesis [92]. Moreover, usnic acid can inhibit multidrug resistance (MDR) efflux pumps, reducing the ability of Methicillin-Resistant *S. aureus* to expel antibiotics and thereby increasing its sensitivity to drugs such as vancomycin and norfloxacin; it disrupts pre-formed biofilms and inhibits the formation of new ones by reducing surface attachment and inhibiting bacterial quorum sensing [92,93]. Investigating the inhibitory potential of dry *U. barbata* extracts against different *Staphylococcus* sp, Idamokoro et al. obtained MIC values ranging from 0.04 to 10 mg/mL for the methanol extract, and from 0.16 to 5 mg/mL for the ethyl acetate extract [94]. Our MIC value for UBPJO (18.75 mg/mL) is slowly increasing, possibly due to the preparation method for *U. barbata* extract and the selected ratio of dry lichen to oil mixture used as the extraction solvent (Section 4.2).

Our previous research reported a strong antibacterial and antibiofilm activity of Peppermint essential oil from Fares S.A. against *S. aureus* [95]. The results align with those of other studies from the scientific literature [96,97]. Thus, PEO demonstrates significant potential as an antibacterial and anti-biofilm agent against *S. aureus.* PEO appears to cause irreversible damage to the cell membranes, as evidenced by increased membrane permeability and leakage of nucleic acids, proteins, and ATP. Additionally, PEO can inactivate mature *S. aureus* biofilms, highlighting its promising role in managing bacterial infections and biofilm-related challenges [31]. UBPJO is rich in pulegone (41.66%) and isomenthone (19.50%); both are very effective against *S. aureus* and act through a common mechanism of terpenoids [98,99,100]. Jojoba oil could also contribute to the inhibitory activity against *S. aureus*, according to Al-Ghamdi et al. [101].

According to previously published studies, several key mechanisms of action of usnic acid against *C. albicans* have been identified [102,103]. Rankovic et al. reported antifungal activity of dry *U. barbata* acetone extract against *C. albicans* at 6.25 mg/mL [44]. Our MIC for UBPJO is slightly greater, 9.62 mg/mL. Usnic acid reduces the biomass and thickness of mature biofilms, reducing metabolic activity in sessile cells, restricting the transformation from yeast to hyphae (a critical stage in *C. albicans* infection and virulence), inducing both intracellular and extracellular reactive oxygen species, leading to cell damage, lowering the sugar content within the biofilm’s protective exopolysaccharide layer, and showing efficacy against azole-resistant C. *albicans* [103].

The lipophilic properties of PEO, particularly its primary component, menthol, facilitate its integration into the fungal phospholipid bilayer, inducing significant alterations in membrane fluidity and permeability and leading to the leakage of essential intracellular constituents [104]. PEO has also demonstrated the capability to completely inhibit *C. albicans* biofilm formation, thereby preventing the growth and survival of these pathogenic yeast strains [105].

## 4. Materials and Methods

### 4.1. Materials

Jojoba oil (JO) obtained by cold pressing *Simmondsia chinensis* seeds and Vitamin E were supplied by Fagron Hellas (Trikala, Greece). Jojoba oil is highly pure and suitable for cosmetic applications. Its liquid wax composition, primarily long-chain esters, provides oxidative stability, a non-greasy texture, and compatibility with human skin. In our experiment, it was combined with 10% Vitamin E.

Peppermint essential oil (PEO) was purchased from Fares S.A., Orastie, Romania; its CG-MS analysis was previously reported [38]. It was diluted in JO (carrier oil) mixed with 10% Vitamin E to 5% concentration (PJO).

*U. barbata* lichen was harvested in March 2024 from the Călimani Mountains, Romania (47°28′ N, 25°13′ E, at an altitude of 900 m). The freshly collected lichen thalli were separated from impurities, then dried at 18–25 °C in an herbal room, protected from sunlight. Dried lichen preservation for an extended period was performed in similar conditions. It was identified by the Department of Pharmaceutical Botany of the Faculty of Pharmacy at Carol Davila University of Medicine and Pharmacy using standard methods. A voucher specimen is maintained in the Herbarium of the Pharmacognosy Department, Faculty of Pharmacy, Carol Davila University of Medicine and Pharmacy (UBL 3/2024, Ph-UMFCD) [29].

All chemicals, solvents, and reagents were of analytical grade.

### 4.2. Preparation of U. barbata Oil Extract

The oil extract was prepared by cold maceration, which preserves the integrity of bioactive compounds and prevents thermal degradation. The harvested lichen samples were ground and passed through successive 2.5 mm sieves (DIN 1171) and 1.2 mm mesh (DIN 117) for homogenization. Almost 20 g of this mass was accurately weighed using a Kern analytical balance, placed in a 1000 mL brown glass container, and 500 mL of JO enriched with 5% PEO and 10% Vitamin E was added. The sample was macerated for 3 months in a light-protected location at a constant temperature (21–22 °C) [26].

The brown container with both components was manually shaken daily for three months; after this period, the oil extract (UBPJO) was filtered into a brown vessel with a sealed plug and preserved in a plant room, sheltered from sunlight [26].

### 4.3. Antioxidant Activity

#### 4.3.1. DPPH Method

We tested the radical-scavenging capacity of the UBPJO using the 2,2-diphenyl-1-picrylhydrazyl (DPPH) method, as previously described [106,107]. The DPPH method is based on the discoloration of a solution containing the DPPH free radical (Sigma-Aldrich, USA) upon interaction with antioxidants present in the tested sample. For each sample, several dilutions in methanol were prepared (1:1, 1:5, 1:10, and 1:25) to assess the dose dependency of the antioxidant effect.

Each sample was used at a 1:2 ratio with the DPPH working solution, which had an initial OD (optical density) of approximately 0.8.

We performed a kinetic assessment, recording the decrease in OD at 517 nm over 1 h. Also, we conducted endpoint measurements after an incubation period of 5, 30, and 60 min respectively, analyzing the decrease in OD:

ΔOD (%) = 100 × (OD_DPPH_ − OD_DPPH+sample_)/OD_DPPH_.

This was considered to express the antioxidant effect of the tested oil samples.

#### 4.3.2. ABTS Method

We used an ABTS (2,2′-Azino-bis(3-ethylbenzothiazoline-6-sulfonic acid))-based method to assess the antioxidant capacity of the extract [106]. This method is based on measuring the sample’s scavenging capacity of the ABTS+∙ free radical. The stock reagent containing ABTS 7 mM and K_2_S_2_O_8_ 2.45 mM was kept in the dark for 16 h at 4 °C for the generation of the green ABTS+∙ free radical. The working solution (WS) was obtained by diluting the stock with distilled water until an appropriate OD (0.8) was reached at 714 nm.

Samples (undiluted as well as 1:1, 1:5, 1:10, and 1:25 dilutions) were incubated with ABTS WS in a 1:3 ratio, and OD was measured at 714 nm after an incubation period of 10 min. A blank was prepared using methanol instead of the sample. Results are presented as the % decrease in optical density (ΔOD):

ΔOD (%) = 100 × (OD_blank_ − OD_sample_)/OD_blank_.

These are directly correlated to the antioxidant capacity of the samples.

### 4.4. Sunscreen Properties

1 mL of each oil sample was added to a 100 mL volumetric flask and diluted with ethanol to the mark. Further, it was ultrasonicated for 5 min. The sun protection factor (SPF) of the oil samples was determined using a Perkin-Elmer Lambda 35 UV-Vis spectrophotometer (Perkin-Elmer Inc., Waltham, MA, USA) in transmission mode. Oil samples were placed in microcuvettes with a 10 mm light path, and absorbance spectra were recorded over 290–320 nm at 5 nm intervals, with three determinations at each point. SPF values were calculated according to established protocols using the Mansur formula [43] (Equation (1)).

(1) $$SPF=CF\times \sum_{320}^{290}EE\left(\lambda \right)\times I\left(\lambda \right)\times Abs\left(\lambda \right)$$

where CF is the correction factor (usually, CF = 10), EE(λ) is the erythemogenic effect of radiation at wavelength λ, I(λ) is the intensity of solar light at wavelength λ, and Abs(λ) is the absorbance of wavelength λ by the preparation solution. The value of EE(λ)×I(λ) is a constant [108]. The determinations were made for the stock solution (undiluted sample) and for the following dilutions: 1:10, 1:5, 1:4, 1:2, 1:1.

### 4.5. Antimicrobial Activity

To evaluate the antimicrobial activity of PJO and UBPJO, we determined the minimum inhibitory concentration (MIC) and the adhesion capacity of the strains to an inert substrate (ACIP = adhesion capacity inhibition percentage), using the following reference strains purchased from American Type Culture Collection (ATCC): *Staphylococcus aureus* ATCC 25923, *Escherichia coli* ATCC 25922, and *Candida albicans* ATCC 10231.

#### 4.5.1. Determination of Minimum Inhibitory Concentrations

Suspensions with an optical density of 0.5 McFarland for bacteria and 1 McFarland for fungi were prepared, and the following were distributed in the 96-well plates: 100 μL of TSA (Tryptic Soy Agar, Sigma-Aldrich Merck (Dartmand, Germany) liquid culture medium (for bacteria) and RPMI 1640 (American Biorganics, Buffalo, NY, USA) (for fungi), 100 μL of a 100 mg/mL solution of the substances investigated in the first well, after which a binary serial dilution scheme was performed up to well 10, and 20 μL of suspension from the strains to be analyzed, from well 1 to well 11 (11 representing the positive growth control, and 12 representing the negative control). The samples were processed in duplicate. The absorbance readings were performed at 620 nm using a Thermo Scientific™ Multiskan™ GO Microplate Spectrophotometer (Thermo Fisher Scientific, Waltham, MA, USA) [109].

#### 4.5.2. Evaluation of the Influence of Oil Samples on Microbial Adherence Capacity to the Inert Substratum

The influence of UBPJO and PJO on biofilm formation is evaluated using the previously described method to determine MIC values. After reading these values, the degree of adhesion to the inert substrate of the tested strains is evaluated by following these steps: fixation with methanol, staining with 1% crystal violet solution resuspended in 33% acetic acid.

The capacity of adherence inhibition percentage (ACIP) was determined using the following formula:

ACIP (%) = (A_s_ − A_blank_) × 100/(A_c_ − A_blank_)

where A_s_ is the absorbance at 490 nm of the tested samples, and A_c_ is the absorbance at 490 nm of the control [109].

### 4.6. Data Analysis

Almost all measurements were performed in triplicate to ensure reproducibility, and the results are expressed as mean ± standard deviation. Data analysis was performed using XLSTAT Premium v.2025.2.0.1232 (Lumivero, Denver, CO, USA) and Microsoft Excel v. 16.0 19328 (Microsoft Corporation, Redmond, WA, USA). ANOVA single factor was used to detect significant differences between variables (*p* < 0.05) [95].

## 5. Conclusions

The present study investigated the antioxidant, photoprotective, and antimicrobial properties of a complex oil lichen extract, *U. barbata,* in Jojoba oil enriched with 5% Peppermint essential oil and 10% Vitamin E, with potential applications in the cosmetic field. The results suggest synergy between the active ingredients across all evaluated pharmacological activities. UBPJO offers promising perspectives for the further development of multifunctional formulations that could protect the skin against oxidative stress and UV radiation, maintain hydration, and support skin health through antioxidant and antimicrobial properties.

In the future, advanced studies may focus on incorporating UBPJO into various cosmetic formulations to investigate its properties and valorize its benefits in a skincare routine or in the therapy of various skin conditions.

## Figures and Tables

**Table 1:** Contributions and roles of all four constituents of *U. barbata* oil extract, and the main phytoconstituents responsible for UBPJO pharmacological properties.

Ingredient	Primary Contribution	Role	References
**Jojoba Oil**	Carrier, emollient, skin barrier support	Stable lipid matrix that protects and delivers other actives	[27]
**Peppermint Oil (5%)**	Antimicrobial, cooling, antioxidant	Adds volatile compounds; enhances penetration via cooling/vasodilatory effect	[28,29,30,31,32,33,34]
**Vitamin E (10%)**	Lipid-soluble antioxidant, photoprotection	Regenerates other antioxidants; protects unsaturated components from oxidation	[35,36]
***U. barbata* extract**	Phenolic-rich, antioxidant, antimicrobial, and potential UV-filter	Strongly boosts TPC; extends oxidative stability; adds lichen-specific bioactive metabolites	[37]
**Main constituents**	**Samples**		
	**PJO** [26]		**UBPJO** [26]
TPC (µg GAE/g)	247.56		297.27
***Volatile constituents (%)***	**PEO** [38]	**PJO** [26]	**UBPJO** [26]
L-Limonene	2.08	9.93	7.11
(+/−)-Linalool	0.06	0.11	-
L-menthone	-	3.94	2.97
(−)-isomenthone/cis-p-Menthan-3-one	26.52	18.34	19.50
Methyl chavicol/estragole	-	8.99	7.81
trans-Carveol	-	1.10	0.80
Neoisomenthol	55.09	-	-
Eucalyptol	5.04	-	-
(+)-Pulegone	-	29.93	41.66
Carvone	-	0.64	1.38
8-Hydroxy-p-menthan-3-one	-	1.34	1.62
Limonene-1,2-diol/Limonene glycol	-	1.33	0.52
Methyleugenol	-	1.63	0.95
trans-Caryophyllene	1.61	0.81	0.51

TPC—Total phenolic content; PJO—Jojoba oil with 5% Peppermint Essential Oil and 10% Vitamin E; UBPJO—*U. barbata* oil extract.

**Table 2:** The results for PJO absorption using the spectrophotometric method.

λ (nm)	EE(λ) × I(λ)	Abs(λ)					
		Dilution 1 (1:10)	Dilution 2 (1:5)	Dilution 3 (1:4)	Dilution 4 (1:2)	Dilution 5 (1:1)	Stock Solution
290	0.0150	0.05738	0.14074	0.23101	1.11833	3.15666	3.34523
295	0.0817	0.04874	0.13292	0.21940	1.11931	3.14826	3.29647
300	0.2874	0.04477	0.12791	0.21074	1.11840	3.16249	3.17902
305	0.3278	0.04199	0.12178	0.20172	1.10897	3.14510	3.08016
310	0.1864	0.03879	0.11660	0.19207	1.10553	3.12180	2.89896
315	0.0837	0.03684	0.11372	0.18607	1.10798	3.11090	2.72582
320	0.0180	0.03630	0.11508	0.18355	1.12044	3.06927	2.62935

EE(λ)—the erythemogenic effect of radiation at wavelength λ; I(λ)—is the intensity of solar light at wavelength λ; Abs(λ)—the absorbance of wavelength λ by the preparation solution; PJO—Jojoba oil with 5% Peppermint Essential Oil and 10% Vitamin E.

**Table 3:** The results for UBPJO absorption using the spectrophotometric method.

λ (nm)	EE(λ) × I(λ)	Abs(λ)					
		Dilution 1 (1:10)	Dilution 2 (1:5)	Dilution 3 (1:4)	Dilution 4 (1:2)	Dilution 5 (1:1)	Stock Solution
290	0.0150	0.12076	0.36644	0.50656	1.41246	3.36631	3.26499
295	0.0817	0.08799	0.30897	0.42907	1.29734	3.36670	3.30553
300	0.2874	0.05972	0.25759	0.36068	1.19441	3.23669	3.21629
305	0.3278	0.04005	0.21727	0.30788	1.10855	3.29269	3.27829
310	0.1864	0.02817	0.18461	0.26462	1.05207	3.27292	3.21496
315	0.0837	0.02332	0.16505	0.23617	1.00143	3.12497	3.19270
320	0.0180	0.02253	0.14380	0.21445	0.97780	3.19647	3.16719

EE(λ)—the erythemogenic effect of radiation at wavelength λ; I(λ)—is the intensity of solar light at wavelength λ; Abs(λ)—the absorbance of wavelength λ by the preparation solution; UBPJO—*U. barbata* oil extract.

**Table 4:** Minimum inhibitory concentration (MIC) values.

Microbial Strain	MIC (mg/mL)			MIC (µg/mL)
	Positive Control	Oil Samples		* Antimicrobial Drug Streptomycin/Ketoconazole [44]
	T80	PJO	UBPJO	
*Staphylococcus aureus* ATCC 25923	50 ± 0.00 ^a^	37.50 ± 0.00 ^a^	18.75 ± 6.25 ^a^	31.25 [44]
*Escherichia coli* ATCC 25922	37.50 ± 12.50	50 ± 0.00	50 ± 0.00	31.25 [44]
*Candida albicans* ATCC 10231	50 ± 0.00 ^b^	37.50 ±12.50 ^c^	9.62 ± 2.87 ^b,c^	1.95 [44]

MIC values are expressed as mg/mL for samples and T80 and as µg/mL for conventional antibiotics [44]. T80 = Tween 80 20% in ethanol; PJO = Jojoba oil enriched with 5% Peppermint oil and 10% vitamin E; UBPJO = *U. barbata* extract in PJO. The differences between values (mg/mL) noted with the same superscript letter in the same row are statistically significant. * Antimicrobials: Streptomycin (for *S. aureus* and *E. coli*) and Ketoconazole (for *C. albicans*) [44].

## Data Availability

The original contributions presented in this study are included in the article. Further inquiries can be directed to the corresponding authors.

## Abbreviations

The following abbreviations are used in this manuscript:

AFM	Atomic Force Microscopy
FTIR	Fourier Transform Infrared spectroscopy
PEO	Peppermint essential oil
JO	Jojoba oil
PJO	Jojoba oil enriched with 5% PEO and 10% Vitamin E
UBPJO	*Usnea barbata* extract in PJO
DPPH	2,2-diphenyl-1-picrylhydrazyl
ABTS	2,2′-azino-bis(3-ethylbenzothiazoline-6-sulfonic acid)
UHPLC	Ultra High-Performance Liquid Chromatography
GC-MS	Gas Chromatography—Mass Spectrometry
OD	Optical density
ΔOD	Decrease in optical density
SPF	Sun Protection Factor
ACIP	Adhesion capacity inhibition percentage
MIC	Minimum inhibitory concentration
ROS	Reactive oxygen species
TRPM8	Transient Receptor Potential Cation Channel Subfamily M Member 8
TNF	Tumor Necrosis Factor-Alpha
